# The function of 7D-cadherins: a mathematical model predicts physiological importance for water transport through simple epithelia

**DOI:** 10.1186/1742-4682-8-18

**Published:** 2011-06-10

**Authors:** Mareike Ahl, Agnes Weth, Sebastian Walcher, Werner Baumgartner

**Affiliations:** 1 Department of Cellular Neurobionics, Institute of Zoology, RWTH-Aachen University, Aachen, Germany; 2 Department of Mathematics I, RWTH-Aachen University, Aachen, Germany; 3 Lehrstuhl A Mathematik, RWTH-Aachen University, Aachen, Germany

## Abstract

**Background:**

7D-cadherins like LI-cadherin are cell adhesion molecules and represent exceptional members of the cadherin superfamily. Although LI-cadherin was shown to act as a functional Ca^2+^-dependent adhesion molecule, linking neighboring cells together, and to be dysregulated in a variety of diseases, the physiological role is still enigmatic. Interestingly 7D-cadherins occur only in the lateral plasma membranes of cells from epithelia of water transporting tissues like the gut, the liver or the kidney. Furthermore LI-cadherin was shown to exhibit a highly cooperative Ca^2+^-dependency of the binding activity. Thus it is tempting to assume that LI-cadherin regulates the water transport through the epithelium in a passive fashion by changing its binding activity in dependence on the extracellular Ca^2+^.

**Results:**

We developed a simple mathematical model describing the epithelial lining of a lumen with a content of variable osmolarity covering an interstitium of constant osmolarity. The width of the lateral intercellular cleft was found to influence the water transport significantly. In the case of hypertonic luminal content a narrow cleft is necessary to further increase concentration of the luminal content. If the cleft is too wide, the water flux will change direction and water is transported into the lumen. Electron microscopic images show that in fact areas of the gut can be found where the lateral intercellular cleft is narrow throughout the lateral cell border whereas in other areas the lateral intercellular cleft is widened.

**Conclusions:**

Our simple model clearly predicts that changes of the width of the lateral intercellular cleft can regulate the direction and efficiency of water transport through a simple epithelium. In a narrow cleft the cells can increase the concentration of osmotic active substances easily by active transport whereas if the cleft is wide, friction is reduced but the cells can hardly build up high osmotic gradients. It is now tempting to speculate that 7D-cadherins, owing to their location and their Ca^2+^-dependence, will adapt their binding activity and thereby the width of the lateral intercellular cleft automatically as the Ca^2+^-concentration is coupled to the overall electrolyte concentration in the lateral intercellular cleft. This could provide a way to regulate the water resorption in a passive manner adapting to different osmotic conditions.

## Background

Epithelia cover inner and outer surfaces of the body, thus they represent the primary barrier for controlled transport of water or dissolved molecules into or out of the body. For this barrier to be efficient the adhesion between neighbouring epithelial cells is vital [1].

Adhesive contacts between adjoined cells play a crucial role in various physiological and pathophysiological aspects of tissue organization, differentiation, and function. The important biological and medical aspects of such stable intercellular adhesions are well established [1]. In cellular monolayers that form permeability barriers like the simple epithelial lining of the intestine or the renal tubuli, adhesion between cells is mainly accomplished by the junctional complex. This junctional complex consists of the tight junction (TJ, zonula occludens), the adherens junctions (AJ, zonula adherens) and the desmosomes (macula adhderens). The TJs are mainly composed of a branching network of sealing strands, each strand is formed from a row of transmembrane proteins of both cell membranes with the extracellular domains joining directly [2]. The major types of these proteins are the claudins and the occludins. The TJ are responsible for the sealing of the lateral intercellular cleft and for allowing a selective transport of water or small molecules in a controlled way.

The AJs are mainly composed of cadherins, single membrane spanning, Ca^2+^-dependent glycoproteins interacting with the cadherins of adjoined cells. These junctions are mainly responsible for the mechanical strength of the junctional complex. Moreover the desmosomes are also responsible for mechanical strength, forming spot-like interaction sites randomly arranged on the lateral sides of plasma membranes composed of desmocadherins, a specialised family of cadherins.

In addition to the above described junctions and the corresponding adhesion molecules, in recent years a distinct group within the cadherin superfamily denoted as 7D-cadherins (7 Domain cadherins) [3] was found. The LI- (Liver Intestine-) cadherin, which is expressed in polarized epithelial cells of liver and intestine [4,5] was the first identified member of this family. Later another member of this group, the Ksp-cadherin, was identified in the kidney [6]. LI-cadherin is uniformly distributed along the lateral contact zones but is excluded from adherens junctions or desmosomes [4], whereas the coexpressed classical cadherins or desmocadherins are concentrated in these specialized membrane regions [7]. In contrast to classical cadherins the 7D-cadherin is composed of seven extracellular cadherin repeats and its very short cytoplasmic domain shows no similarity to the highly conserved cytoplasmic region of classical cadherins necessary for the interaction with catenins and thus with the cytoskeleton [8]. Although LI-cadherin was shown to act as a functional Ca^2+^-dependent adhesion molecule [9,10] and to be dysregulated in a variety of diseases [11-14], the physiological role is still enigmatic.

It is worth noting that the above mentioned 7D-cadherins are expressed in epithelia which are involved in water resorption under different osmotic conditions. In the intestine and colon for example water has to be reabsorbed from the chymus in order to avoid water loss. The luminal content of the gut shows osmolarities from almost pure water to the high osmolarity of the faeces which is far above the physiological osmolarity of the interstitium which is about 300 mM [15]. The situation in the kidney or in the liver, where the urine or the bile are to be produced, is similar. In all these organs water transport plays an important role. Thus it is tempting to assume the involvement of 7D-cadherins in the regulation of water absorption.

A second noteworthy point is the unusual Ca^2+^-dependency of the LI-cadherin function. As we could show recently, the LI-cadherin mediated adhesion becomes absolutely insufficient at Ca^2+^-levels that are only slightly below the physiological level of about 1.5 mM [10]. This is in contrast to classical cadherins which can tolerate Ca^2+^-levels down to 0.3 mM [16-19].

These two facts that i) 7D-cadherins are expressed in epithelia where water transport under different osmotic conditions takes place and ii) that LI-Cadherin displays an extreme sensitivity towards decreased Ca^2+^-levels led us to the development of a model for the water resorption in epithelia. We took into account that due to viscous friction a small pressure gradient will be built up in the lateral intercellular cleft (LIC) between epithelial cells during water transport. Our hypothesis is that in the case of hypotonic medium in the lumen of the resorbing organ (e.g. the gut lumen), a wide cleft facilitates water transport because of friction minimisation. On the other hand, if the medium is hypertonic, i.e. exhibits high osmolarity, a narrow intercellular cleft favours water resorption since in the small volume, an osmotic gradient between the lumen and the lateral intercellular cleft can be built up by ATPases, thus allowing for water uptake from the lumen even if the content, e.g. the faeces, exhibits osmolarity far above the isotonic electrolyte concentration. The derived simple theoretical model shows interesting effects in support of the above hypothesis and suggests a role for 7D-cadherins in the regulation of osmotically driven water transport.

## Results

### Model for water transport through epithelial monolayers

**Figure 1:**
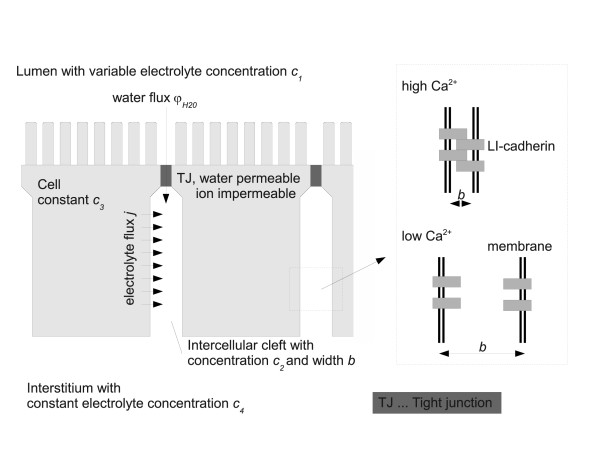
**Model for water and electrolyte transport through simple epithelia**. The model comprises four compartments which are (1) the lumen of the organ (e.g. the gut), (2) the lateral intercellular cleft (LIC) which is assumed to be homogeneous with respect to electrolyte concentrations, (3) the cytoplasm of the cell and (4) the interstitium. In the lumen a given concentration of electrolytes is assumed. The tight junctions (TJ) separate the lumen (1) and the LIC (2) and are assumed to be impermeable for the electrolyte and permeable for water with a permeability coefficient *K_TJ_*. The concentration of electrolytes in the cytoplasm is assumed to be constant *c_3_*. ATPases are assumed to pump the electrolyte through the lateral membrane into the lateral intercellular cleft. The interstitium is assumed to display a constant electrolyte concentration *c_4 _*which is maintained through the blood vessels located here. The important compartment is the LIC. Water enters this compartment through the TJ or from the interstitium. Ions enter through the lateral membrane due to the ATPases and leave the LIC due to diffusion and due to the water flux *φ_H2O_*, flushing the lateral intercellular cleft. The width of the lateral intercellular cleft b is dependent on the binding activity of the 7D-cadherins, which in turn is dependent on the extracellular Ca^2+^-level.

The model, which follows in principle the approach of a so called standing osmotic gradient [2,15,20], is depicted in Figure 1. It comprises four compartments, viz. (1) the lumen of the organ (e.g. the gut), (2) the lateral intercellular cleft (LIC) which is assumed to be homogeneous with respect to electrolyte concentrations, (3) the cytoplasm of the cell and (4) the interstitium. In the lumen a given concentration of electrolytes is assumed which may vary with the position in the gut from highly hypertonic to hypotonic. For our model we do not take into account the exact ion composition of the electrolyte solution in the different compartments but rather assume one osmotic active electrolyte. The tight junctions (TJ) separate the lumen (1) and the LIC (2). For simplicity assume the TJ to be impermeable for the electrolyte and permeable for water with a permeability coefficient *K_TJ_*. As we use a compartment model, this assumption and the following assumption for the plasma membrane to be impermeable for water but permeable for ions will change the described results only quantitatively but not qualitatively. Thus we expect a water flux *φ_H2O _*through the TJ due to a difference in the combined osmotic and hydrostatic pressure, i.e.(1)

With *K_TJ _*being the hydraulic conductivity of the TJ*, R *is the gas constant and *T *the absolute temperature. Thus *RT(c_2_-c_1_) *describes the osmotic pressure difference. ζ is a viscous friction coefficient in the cleft. Thus ζ/*b*^2^·*φ_H2O _*is the hydrostatic pressure difference that occurs due to the water flux in the LIC. The inverse square dependence on the cleft width is the simplest model describing the most conservative approach. A higher power of *b *would yield even more pronounced results as will be discussed later. For simplicity we assume the plasma membrane to be impermeable for water, therefore the water flux is only maintained through the tight junctions.

The concentration *c_3 _*of electrolytes in the cytoplasm is assumed to be constant. This is reasonable as there is controlled ion transport from the lumen and from the basal plasma membrane to maintain isoosmolarity for the cell under any circumstances. ATPases are assumed to pump the electrolyte through the lateral membrane into the LIC. For simplicity we assume the lateral ion flux *j *to be constant through the whole lateral membrane and to be independent of the concentrations *c_2 _*(thus there is no transport from the cleft into the cell), and proportional to the concentration *c_3 _*which is assumed constant in our model. As will become evident later on, this assumption is a conservative one which would cause an underestimating of the effects that will be shown below. The electrolyte concentration *c_4 _*in the interstitium is assumed to be constant, being maintained through the blood vessels located here. The important compartment is the LIC. Water enters this compartment through the TJ or from the interstitium. Ions enter through the lateral membrane due to the ATPases and leave the LIC due to diffusion and due to the water flux *φ_H2O_*, i.e. through convective flow of the ions. Thus we have an electrolyte flux from the cell into the LIC(2)

with *j *being the flux density and *A *being the area of the lateral membranes; and we have an electrolyte flux out of the LIC into the interstitium which equals(3)

Here the first term describes the diffusion out of the cleft into the interstitium with  being the over all diffusion coefficient of the electrolyte. The second term describes the above mentioned convective flow of ions due to the water flux.

The change of the electrolyte concentration in the LIC, according to the law of mass conservation, equals the sum of the inward and outward electrolyte fluxes divided by the volume of the LIC(4)

Substituting Eq. 1 to Eq. 3 in Eq. 4 and introducing the abbreviations(5)

we obtain a differential equation for the concentration *c_2_*, namely(6)

This is an ordinary differential equation of Riccati type which could be solved in principle. However, we are interested in the positive equilibrium solution only, to which every solution with positive initial data converges, i.e. we consider the solution of dc_2_/dt = 0. The stationary concentration in the LIC turns out to be(7)

Solving Eq. 7 allows for the determination of all concentrations and fluxes, especially *φ_H2O _*in our system in dependence on the luminal electrolyte concentration and the width of the LIC.

From Eq. 1 we can directly conclude that the direction of the water flux *φ_H2O _*depends on the sign of *(c_2_-c_1_)*. Rearranging Eq. 6 for the stationary case we obtain the equation(8)

Note that the first factor on the left-hand side is always negative. The water flux changes the direction if  changes sign, and therefore if the right-hand side of Eq. 8 changes sign. Thus we obtain the concentration in the lumen at which the water flux changes the direction by setting  equal zero in Eq. 8. This leads to(9)

with  denoting the luminal concentration at which the water flux changes direction. As is obvious from Eq. 1, the water flows from the lumen into the interstitium if *c_1 _*= 0. If the osmolarity of the luminal content increases, flux decreases and at the luminal concentration  the water flux becomes zero. If *c_1 _*is further increased, the water flows from the interstitium into the lumen. One should keep in mind here that, depending on the parameters,  may be too high to be of physiological relevance.

### Influence of 7D-cadherin binding onto the water transport

**Figure 2:**
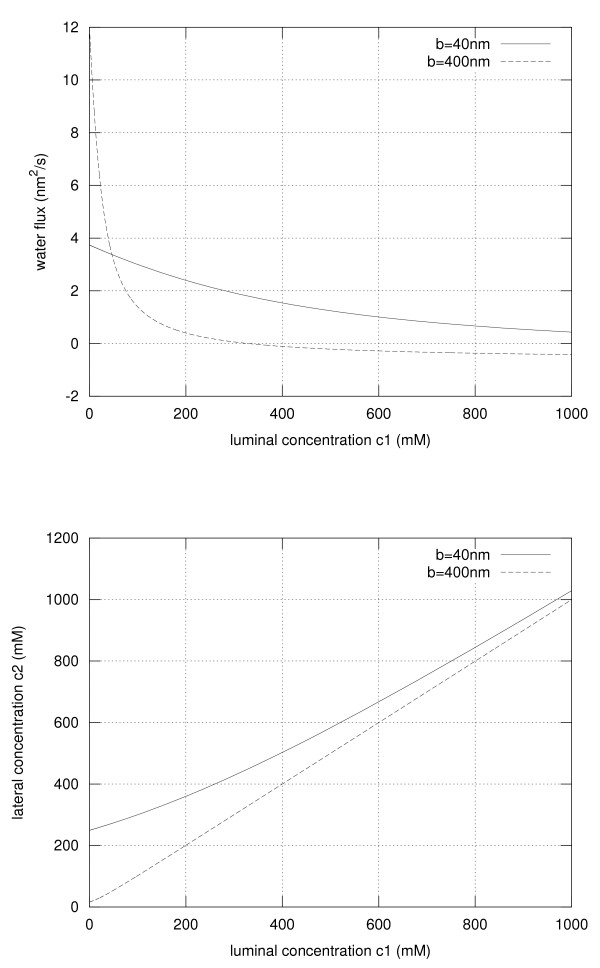
**Water flux and electrolyte concentration in the lateral intercellular cleft**. The water flux through the TJ and thus through the lateral intercellular cleft (upper panel) and the electrolyte concentration *c_2 _*(lower panel) are depicted in dependence on the luminal electrolyte concentration *c_1_*. The results are shown for LI-cadherin binding, i.e. a narrow intercellular cleft (solid line) and for inactive LI-cadherin, i.e. a wide intercellular cleft (dashed line).

The width of the lateral intercellular cleft *b *is dependent on the binding activity of the 7D-cadherins, which in turn is dependent on the extracellular Ca^2+^-level. This is depicted in Figure 1. Typical values for the various parameters needed for our model are shown in table 1. Based on these physiological parameters, which are taken from different studies, we could calculate the concentration *c_2 _*and the water flux *φ_H2O _*in dependence of the luminal concentration and on the width of the LIC. The results are depicted in Figure 2. Clearly the width has a dramatic effect on the concentration *c_2 _*and on the water flux. As expected for hypotonic conditions in the lumen, i.e. for a low electrolyte concentration, a wide intercellular cleft (*b *= 400 nm) leads to a higher water flux when compared to the narrow cleft (*b *= 40 nm) as the friction is reduced and the osmotic gradient can be maintained by diffusion of the electrolyte from the interstitium into the cleft. The concentration *c_2 _*follows very much the luminal concentration, i.e. *c_2_*≈*c_1_*. However, under hypertonic conditions the water flux is inverted, i.e. water flows from the interstitium into the lumen if the cleft is 400 nm wide. Notably this is not the case if the cleft is narrow. For *b *= 40 nm the volume of the lateral intercellular cleft is small, leading to a concentration *c_2 _*significantly higher than the luminal concentration due to the electrolyte flux *j *maintained by the ATPases. Under these conditions the osmotic gradient is still directed from the lumen into the cleft allowing to further increase the osmolarity of the luminal content.

**Table 1:** Parameter values

parameter	symbol	value	reference
luminal electrolyte concentration	*c_1_*	0-1000 mM	[2,15,20]

interstitial electrolyte concentration	*c_4_*	300 mM	[1,2,15]

ion flux through the lateral membrane	*j*	18.5 × 10^-6 ^mmol/s/cm	[1,15]

height of the epithelial cell	*h*	100 μm	[2,15,20]

water conductivity of the tight junction	*K_TJ_*	0.5 cm/cm/mmHg	[1,2]

gas constant times room temperature	*RT*	4500 J/mmol	[22]

diffusion coefficient		50 nm/s	[22]

friction coefficient	ζ	0.1-10 kg/s	[2]

**Figure 3:**
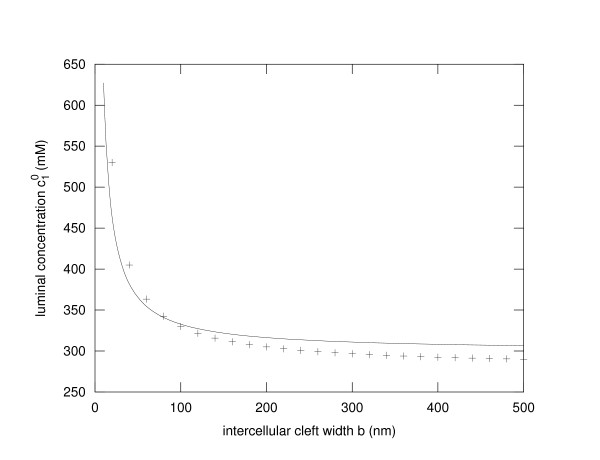
**Critical luminal electrolyte concentration**. The critical electrolyte concentration , i.e. the luminal electrolyte concentration at which the water flux through the tight junction changes sign is depicted in dependence on the width of the lateral intercellular cleft *b*. The solid line represents the results of Eq. 9. As evident from this equation a 1/*b *dependence of  can be observed. The + - signs show the results from a full numerical simulation of a finite volume model taking various additional effects into account (see additional file 1). Clearly the principal dependency is highly similar.

The critical concentration , i.e. the luminal concentration at which the water flux changes its direction is depicted in Figure 3. The solid line shows the behaviour according to Eq. 9. The results of a finite volume numerical simulation (see additional file 1) that takes into account different additional effects like a finite ion permeability of the TJ and a certain water permeability of the plasma membrane as well as a barrier function of the basal membrane, is shown as +-signs. Although there are quantitative differences, the principal behaviour, a 1/b-dependence, is conserved.

### Electron microscopic analysis of the lateral intercellular cleft in the gut epithelium

To check if our model is reasonable, we investigated the lateral intercellular cleft of mouse enterocytes with the transmission electron microscope.

**Figure 4:**
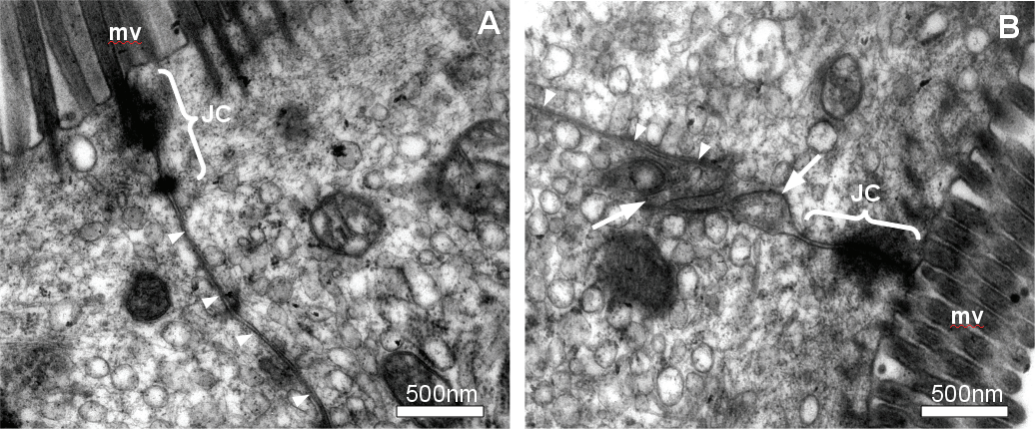
**Intercellular cleft in the mouse gut**. Transmission electron micrographs of enterocytes from different areas of the gut. Clearly there are areas where the lateral intercellular cleft, marked with white arrowheads, is narrow throughout the height of the cell (A) whereas in other areas widening can be observed (B), which are marked with arrows. Junctional complexes (JC) and microvilli (mv) can be observed.

As shown in Figure 4, there are sections of the gut where the LIC is narrow (20-40 nm) throughout the lateral surface of the cells whereas in other regions we find partial widening of this intercellular cleft. Not surprisingly these widenings are not of equal width over the cell height. Although this does not prove our hypothesis we found in samples taken from three different mice areas with and without widening up to 0.5 μm.

## Discussion

We have derived a simple model for the osmotically driven paracellular water transport through simple epithelia. Although the model makes several simplifying assumptions, like the assumption of homogeneous electrolyte concentration throughout the length of the lateral intercellular cleft (LIC), it describes the role of LIC width *b *for water transport very well in a qualitative and reasonably well in a quantitative sense. Therefore it appears to be well suited to explain interesting facts about the influence of the binding of 7D-cadherins like the LI-cadherin. With respect to the change of the direction of the water flux through the tight junction, a comparison with a numerical simulation, taking different additional parameters into account shows, that our simple model describes this phenomenon rather good. It was clearly found that in the case of hypotonic content of the lumen a wide LIC is advantageous as viscous friction is reduced. In the model presented above, a simple Stokes approach was used to take friction into account. If the friction depends on a higher power of the width of the LIC, e.g. because of effects of the glycocalix or proteins or due to water structuring in the cleft, the described effects will be even stronger. In the case of a wide cleft, the electrolyte concentration within the cleft follows pretty much the concentration within the lumen.

On the other hand, if the luminal content is hypertonic, water transport would be inverted in the case of a wide LIC. Only if the LIC is narrow, the ATPases located in the lateral plasma membrane would be able to increase the osmolarity in the LIC so that water is still transported from the lumen into the cleft. From there the hypertonic solution is transported by fluid flow and diffusion into the interstitium where the electrolyte and the water will be taken up by the blood vessels located here.

We varied the parameters of the model within a rather wide range and found only quantitative changes but the qualitative behaviour, i.e. the dependence of the water absorption on the luminal osmolarity in combination with the width of the LIC was unchanged. Moreover, if we considered "improved" versions of the model in order to account for possible oversimplifications, like the lack of permeability for water of the plasma membrane, or the assumed negligibility of the reflection coefficient of the basal membrane (see supplement), we found the same qualitative behaviour.

However, the expressions become much more complicated and the derivation of interesting facts such as the dependence of the critical luminal electrolyte concentration on the cleft width *b *(Eq. 9) becomes much more involved with no gain in clarity. This seems to be one of the (not infrequent) scenarios where, in spite of oversimplifying assumptions, a model still yields useful and realistic information. The described model behaviour led us to the hypothesis that 7D-cadherins might be important for the regulation of water transport through epithelia. As mentioned above, LI-cadherin for example is located all over the lateral plasma membranes in the epithelia whereas the E-cadherin is strictly localised in the adherens junction at the luminal end of the LIC. Desmocadherins are localised in the desmosomes, spot-like adhesive sides, mainly in the more luminal part of the cleft. E-cadherin as well as desmocadherins are much less sensitive to extracellular Ca^2+ ^than LI-cadherin. Thus we would expect, that if Ca^2+ ^is depleted in the case of hypotonic luminal content, the LI-cadherin trans-interactions will be weakened while the adherens junction and the desmosomes are still stable. The hydrostatic pressure that is generated due to the water transport within the cleft will separate the weakened LI-cadherin bounds and thus lead to a widening of the lateral intercellular cleft. The wider cleft provides less viscous friction and thus much higher water flux from the lumen into the interstitium. In our example we obtained an up to three times higher water flux in the wide cleft. If now the osmolarity in the lumen is changed to hypertonic, the water and thus the electrolyte flux will be reversed. Therefore the electrolyte concentration in the LIC will be increased to the levels in the interstitium. Under these conditions the Ca^2+^-levels will rise leading to active 7D-cadherins. If these cadherins bind, the cleft will become narrow, allowing the ATPases to build up an osmotic gradient out of the lumen re-establishing the water transport into the body. A molecular hint might be the fact that these cadherins, compared to classical cadherins, are longer and can therefore be more effective in re-establishing trans-interactions with cadherins of the adjoined cells. The osmotic conditions within the gut are rather complicated as for optimal efficiency of the digestion water has to be transported into the gut and out of the gut depending on the state of digestion. 7D-cadherins might be an elegant means of effectively regulating the water transport. Of course there are other mechanisms too, but the passive reaction of the cadherins to the Ca^2+^-changes that occur coupled to the osmotic changes might be a central and effective way to achieve efficient water transport. Clearly we found by transmission electron microscopy that the LIC width is not uniform throughout the gut. There are areas where the cleft is narrow throughout the height of the cell whereas in other regions we clearly identified widening of the cleft. This is only a clue and no proof. Another clue is the expression pattern of 7D-cadherins. As stated initially, 7D-cadherins are expressed in epithelial cells in the gut, the kidney and in the liver. These organs need for their functions regulated water transport through the epithelia under variable osmotic conditions.

To clearly show the involvement of 7D-cadherins in the regulation of water transport, additional and more sophisticated experiments would be necessary. The water resorption in dependence on the state of the LI-cadherin should be measured. Unfortunately no knock out mouse for LI-cadherin is available yet, which would allow detailed characterisation of the resorption in the gut in dependence on the osmolarity of the luminal content. Comparison with wild type control mice should yield experimental evidence whether or not our model predictions are correct. Alternatively extensive experiments with isolated guts could be done where the water resorption in dependence on the luminal content could be measured followed by TEM-studies of the investigated tissue. If clear correlations of the water uptake - osmolarity relation and the width of the LIC are found, the hypothesis could be accepted. In any case, a closer look at the influence of LI-cadherin onto the water transport is definitely worth spending time and money. Dysregulation of water and electrolyte uptake are known to cause severe physiological problems. Perhaps the 7D-cadherins will prove to be an important target for the medical therapeutic actions in the near future.

## Conclusions

A simple mathematical model predicts that changing the width of the lateral intercellular cleft (LIC) between neighbouring epithelial cells can regulate the direction and efficiency of water transport through a simple epithelium. In a narrow cleft the cells can increase the concentration of osmotic active substances easily by active transport, but the friction of the transported water is high. If the cleft is wide, friction is reduced but the cells can hardly built up high osmotic gradients. As the Ca^2+^-concentration is principally coupled to the overall electrolyte concentration, the activity of 7D-cadherins is presumably strictly coupled to the osmotic conditions in the water absorbing organs. Thus one can assume that active 7D-cadherins, due to their trans-interaction with cadherins of neighbouring cells, will cause a narrowing of the lateral intercellular cleft. 7D-cadherins due to their location and their Ca^2+^-dependence could thus provide a way to passively adapt the direction and efficiency of water transport through epithelia. Experimental studies will be necessary to verify or falsify the proposed hypothesis of the involvement of 7D-cadherin in the regulation of water transport.

## Methods

### Numerical calculations

All calculations were carried out using either MatLab™ (Mathworks) or the the free software Octave on a Pentium 4 PC using Ubuntu "Maverick Meerkat" with a GNU-interface. The finite volume simulation (see additional file 1) was set up similar to an approach described previously [21].

All calculations were carried out assuming the LIC to be infinitely deep. For the analytical model all concentrations and fluxes are assumed to be uniform throughout the depth. For the numerical calculations all results were calculated for a depth of unit size, i.e. of 1 nm depth. Thus the area *A *is given as 1 nm times cell height *h*.

### Transmission electron microscopy

For electron microscopy, mice were anaesthetized using chloroform and killed by cervical dislocation. The gut was immediately removed, washed for 10 s in ice cold HBSS (Sigma) and fixed over night in HBSS containing 4% formaldehyde and 2.5% glutaraldehyde. Then the gut was cut into 5 mm pieces. After rinsing the samples three times in 0.1 M sodium cacodylate (Sigma) containing 7% (w/v) succrose for 10 min on ice, they were rinsed twice in 0.1 M sodium cacodylate and then postfixed in 2% (w/v) OsO_4 _(Sigma) in 0.1 M sodium cacodylate for 2 h on ice. The samples were rinsed again in 0.1 M sodium cacodylate at room temperature and dehydrated in ascending concentrations of ethanol (30% and 40% for 15 min each, 50% for 60 min, 60%, 75%, and 90% for 30 min, 100% overnight, and 100% for 60 min). After dehydration, samples were equilibrated twice in propylene oxide (Serva) for 30 min, followed by 50% (w/v) propylene oxide and 50% (w/v) resign (Epon 812; Serva) overnight. The samples were incubated twice in 100% Epon for 2 h and then embedded in Epon 812. Then ultra thin sections of 90 nm thickness were cut and observed using a Zeiss EM10 TEM.

## Competing interests

The authors declare that they have no competing interests.

## Authors' contributions

MA did most of the calculations and the analytical characterization of the model as well as the literature search for the necessary parameters. AW performed the electron microscopy of the mouse gut. SW characterized the analytical properties of the model and the corresponding differential equations. He wrote parts of the manuscript. WB did the modeling and the derivation of the initial differential equations, did some calculations and characterizations of the analytical model and he wrote most of the manuscript. All authors read and approved the final manuscript.

## Supplementary Material

Additional file 1 **Finite volume approach for water and electrolyte fluxes**. A finite volume approach for the numerical calculation of the concentrations, pressures and fluxes of water and electrolytes within the lateral intercellular cleft is presented. Click here for file
