# Supplementary material for: The function of 7D-cadherins: a mathematical model predicts physiological importance for water transport through simple epithelia
Source: Theor Biol Med Model. 2011 Jun 10;8:18. doi: 10.1186/1742-4682-8-18 (PMC3138449; doi:10.1186/1742-4682-8-18)
Supplement: Additional file 1 — Finite volume approach for water and electrolyte fluxes. A finite volume approach for the numerical calculation of the concentrations, pressures and fluxes of water and electrolytes within the lateral intercellular cleft is presented. [file 1742-4682-8-18-S1.PDF]

To calculate the exact water and ion fluxes the electrolyte concentrations and the pressures, we set up a finite volume model of the system, similarly to the simple analytical model described in the principal manuscript:

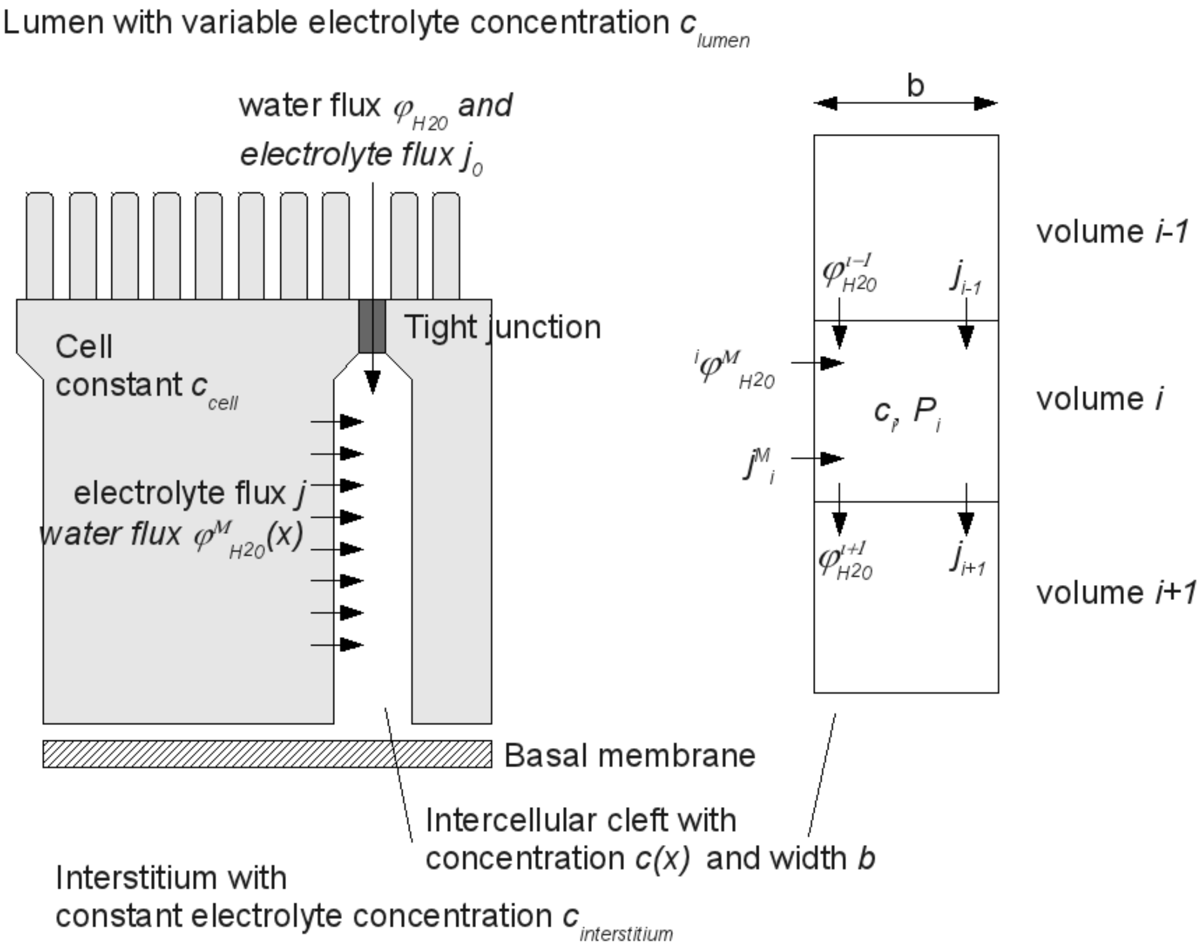

The intercellular cleft is divided into i=1..N (typically N=20) finite volume elements each assumed to have a constant electrolyte concentration c_i and a constant hydrostatic pressure P_i. Water leaved the volume i(i<N) to enter the next volume i+1 and enters the volume i (i>1) from the previous volume i-1 and from the cell through the membrane. The membrane exhibits a certain water permeability maintained e.g. by aquaporin-channels. Similarly ions enter the volume from the neighboring volumes and through the membrane. The ion flux through the membrane is assumed to be constantly maintained by ATPases.

Through the tight junction there are water and ion fluxes into the first volume element i=1. Through the basal membrane there are water and ion fluxes out of the last volume element i=N. Tight junction and basal membrane exhibit a certain water permeability and a certain reflection coefficient for ions. The permeability and conductivity of the basal membrane is typically higher than of the tight junction.

The ion concentrations in the cell and in the interstitium are assumed to be constant c_{cell} and $c_{interstitium}$ respectively. The concentration in the lumen, i.e. c_{lumen} is variable.

The hydrostatic pressure in these compartments is set equal to 0 and the pressures in the finite volumes of the intercellular cleft are to be understood as pressure difference to these compartments.

For each volume element i the surface integral of all water fluxes φ has to vanish as there are no sources or sinks for water in the volumes. The surface integral of all ion fluxes j defines the change rate of the ion concentration times volume. Thus

$$\oiint_{\partial V_i} \varphi \circ dA = 0 \quad \text{and} \quad \oiint_{\partial V_i} j \circ dA = V_i \cdot \frac{\partial c_i}{\partial t}$$

The water and ion fluxes from volume element i into volume element $i+1$ follow to be

$$\varphi^i_{H_2O} = (P_i - P_{i+1}) \cdot \frac{b^2}{\zeta} \quad \text{and} \quad j_i = \varphi^i_{H_2O} \cdot c_i + D \cdot \frac{c_i - c_{i+1}}{\Delta x}$$

i.e. the water flux is driven by the local pressure gradient and follows the Navier-Stokes relation with ζ as the hydraulic friction coefficient and ions are transported by the water flux and by diffusion. In addition to the water and ion fluxes from one volume element into another, water and ions enter the ith volume element through the plasma membrane, i.e.

$$^i\varphi^M_{H_2O} = K_M \cdot [RT(c_i - c_{cell}) - P_i] \quad \text{and} \quad j^M_i = const.$$

This means the water flux through the membrane is osmotically driven and the local hydrostatic pressure acts against the water transport into the intercellular cleft. The pressure in the cell plasma is assumed to be 0. K_M is the water conductivity of the plasma membrane. The ion flux, maintained by ATPases is assumed to be constant.

The water and the ions entering the intercellular cleft from the lumen, i.e. the fluxes into the first volume element (i=1) are given as

$$\varphi^0_{H_2O} = K_{TJ} \cdot [RT(c_{i=1} - c_{lumen}) - P_{i=1}] \quad \text{and} \quad j_0 = \frac{c_{lumen} - c_{i=1}}{R_{TJ}}$$

Here K_{TJ} is the water permeability of the tight junction and R_{TJ} is the reflection coefficient for ions of the tight junction.

Finally the fluxes out of the intercellular cleft into the interstitium over the basal membrane, i.e. the fluxes out of volume element N are given as

$$\varphi^N_{H_2O} = K_{BM} \cdot [RT(c_{i=N} - c_{interstitium}) - P_{i=N}] \quad \text{and} \quad j_N = \frac{c_{i=N} - c_{interstitium}}{R_{BM}}$$

Here K_{BM} is the water permeability of the basal membrane and R_{BM} is the reflection coefficient for ions of the basal membrane.

For stationary conditions, i.e.

$$\frac{\partial c_i}{\partial t} = 0 \quad \forall i \in \{1, .., N\}$$

we obtain for all volume elements, i.e. for all indices $i=1..N$

$$\oiint_{\partial V_i} \varphi \circ dA = 0 \Rightarrow {}^i\varphi^M_{H_2O} \cdot \Delta x + b \cdot (\varphi^{i-1}_{H_2O} - \varphi^i_{H_2O}) = 0 \quad \text{and} \quad \oiint_{\partial V_i} j \circ dA = 0 \Rightarrow j^M_i \cdot \Delta x + b \cdot (j_{i-1} - j_i) = 0$$

Replacing the fluxes by the therms above leads to a system of $2N$ nonlinear equations which can be solved numerically to obtain the unknown variables c_i and P_i defining the physical system.
